# Clinical Outcomes of Acutely Ill Children According to Cycle Threshold Values of Respiratory Viruses Detected by Multiplex PCR Testing

**DOI:** 10.1093/jpids/piad071

**Published:** 2023-09-20

**Authors:** Suvi Mattila, Minna Honkila, Niko Paalanne, Vesa Mäki-Koivisto, Kimmo Halt, Tuomas Jartti, Olli Ruuskanen, Matti Waris, Tytti Pokka, Terhi Tapiainen

**Affiliations:** Department of Pediatrics and Adolescent Medicine, Oulu University Hospital, Oulu, Finland; Research Unit of Clinical Medicine and Medical Research Center Oulu, University of Oulu, Oulu, Finland; Department of Pediatrics and Adolescent Medicine, Oulu University Hospital, Oulu, Finland; Research Unit of Clinical Medicine and Medical Research Center Oulu, University of Oulu, Oulu, Finland; Department of Pediatrics and Adolescent Medicine, Oulu University Hospital, Oulu, Finland; Research Unit of Clinical Medicine and Medical Research Center Oulu, University of Oulu, Oulu, Finland; Clinical Microbiology Laboratory, Nordlab, Oulu, Finland; Department of Pediatrics and Adolescent Medicine, Oulu University Hospital, Oulu, Finland; Research Unit of Clinical Medicine and Medical Research Center Oulu, University of Oulu, Oulu, Finland; Department of Pediatrics and Adolescent Medicine, Oulu University Hospital, Oulu, Finland; Research Unit of Clinical Medicine and Medical Research Center Oulu, University of Oulu, Oulu, Finland; Department of Pediatrics and Adolescent Medicine, Turku University Hospital and University of Turku, Turku, Finland; Department of Pediatrics and Adolescent Medicine, Turku University Hospital and University of Turku, Turku, Finland; Institute of Biomedicine, University of Turku, Turku, Finland; Research Unit of Clinical Medicine and Medical Research Center Oulu, University of Oulu, Oulu, Finland; Research Service Unit, Oulu University Hospital, Oulu, Finland; Department of Pediatrics and Adolescent Medicine, Oulu University Hospital, Oulu, Finland; Research Unit of Clinical Medicine and Medical Research Center Oulu, University of Oulu, Oulu, Finland; Biocenter Oulu, University of Oulu, Oulu, Finland

**Keywords:** cycle threshold values, multiplex polymerase chain reaction, pediatric emergency department, point-of-care testing, respiratory viruses

## Abstract

In this cohort study of 800 children attending a pediatric emergency department at Oulu University Hospital, Finland with fever or respiratory symptoms, the cycle threshold values of point-of-care multiplex polymerase chain reaction testing for respiratory viruses were not associated with hospitalization, respiratory support, or need for intensive care.

## INTRODUCTION

Point-of-care (POC) multiplex polymerase chain reaction (PCR) testing for respiratory pathogens is currently performed in some pediatric emergency departments (EDs) [1]. The PCR cycle threshold (CT) value represents the number of amplification cycles required to yield a positive test result and is inversely correlated with the pathogen load [2]. The test result is commonly given to the clinicians as a negative or positive finding and CT values are not usually reported.

Some correlations between CT values and clinical outcomes have been reported [3, 4]. In respiratory syncytial virus (RSV) infections, high genomic loads have been associated with a higher risk for hospitalization, intensive care unit (ICU) admission and longer hospital stays [4–8]. Higher viral loads in patients with rhinovirus infection have been associated with the risk of admission to the pediatric ICU [3] and a modified response to corticosteroids [9, 10].

We hypothesized that the clinical outcomes of respiratory viral infections may be associated with the detected CT values of respiratory viruses. In this cohort study, the clinical outcomes of acutely ill children were analyzed according to the CT values of respiratory viruses detected by POC multiplex PCR testing at a pediatric ED.

## METHODS

This observational cohort study included children and adolescents aged 0–17 years with fever (≥38.0°C) and/or a respiratory condition or symptom (tachypnea, shortness of breath, apnea, wheezing, cough, rhinitis, croup, sneezing, earache, sore throat, or other suspicions of respiratory infection) at a pediatric ED in Oulu University Hospital, Finland. Patients had participated in the active diagnostic arm of a previous randomized controlled clinical trial [1]. POC multiplex PCR testing results (positive/negative) were given to clinicians at the pediatric ED. The clinicians treating the patients were not aware of the CT values. For research purposes, CT values of the respiratory viruses were obtained from the diagnostic device and manually entered into the statistical software. Patients were recruited in 2019–2020 before the COVID-19 pandemic. The study physicians systematically collected three clinical outcomes from electronic medical records: admission to hospital, need for respiratory support or supplemental oxygen, and admission to a pediatric ICU. The data collection was completed in 2021–2022. The Ethical Committee of the North Ostrobothnia’s Hospital District (EETTMK: 8/2019) approved the study protocol. Written informed consent was obtained from each participant’s legal guardian.

Trained pediatric ED nurses collected the nasopharyngeal swabs by passing a flocked swab (FLOQSwabs, Copan Diagnostics, Inc., California, USA) through the nostril to the nasopharynx, and rotating it three to five times to collect epithelial cells before gently removing the swab. All nurses working in the ED received hands-on training on sample collection before the study commenced. Written instructions for sample collection were provided in the ED. The nurses then directly applied the swab for organism detection to the cartridge of a Respiratory Panel V2 multiplex PCR assay for 18 respiratory viruses or viral subtypes and 3 bacteria analyzing the samples using a QIAstat-Dx™, DiagCORE device. The assay detected the following organisms and subtypes: adenovirus, bocavirus, coronaviruses (HKU1, NL63, OC43, 229E), human metapneumovirus, influenza A (subtype H1N1/2009, H1, H3), influenza B, parainfluenza viruses (1–4), rhinovirus/enterovirus, RSV, *Bordetella pertussis*, *Legionella pneumophila*, and *Mycoplasma pneumoniae*. The samples were tested as dry swab samples (707, 88.4%) or transport medium liquid samples (92, 11.5%). The dry swab samples were analyzed by directly inserting the swab into the cartridge and then breaking the swab shaft, leaving the rest of the swab in the cartridge, as described in the users’ manual. The transport medium liquid samples were tested by placing the swab into the Universal Transport Medium and then loading 300 μl of sample volume from the tube to the cartridge using a single-use transfer pipette.

We used t-test to compare the CT values according to clinical outcomes (admission to hospital, need for respiratory support, and need for intensive care). Then we performed a multivariate logistic regression analysis, adjusted for age and sex, for these clinical outcomes. Analyses were performed using IBM SPSS Statistics for Windows, version 27 (Armond, NY: IBM Corp.).

## RESULTS

We enrolled 829 participants. Organism findings with CT values were available for 800 patients (Supplementary Table 1). The mean age of the children was 3.0 (SD 3.6) years, and 356 (45%) of the participants were girls. Underlying medical conditions were reported in 200 (25%) participants. Altogether, 334 (42%) patients were hospitalized and 50 (6.3%) needed respiratory support or supplemental oxygen.

A respiratory virus was detected in 586 (73%) patients. Two or more respiratory viruses were detected in 106 (13%) patients. The most common viruses were rhino/enterovirus (328, 41%), RSV (133, 17%), and adenovirus (80, 10%). *M. pneumoniae* was detected in 10 (1.3%) patients and *B. pertussis* in 2 (0.3%) patients.

**Figure 1.**
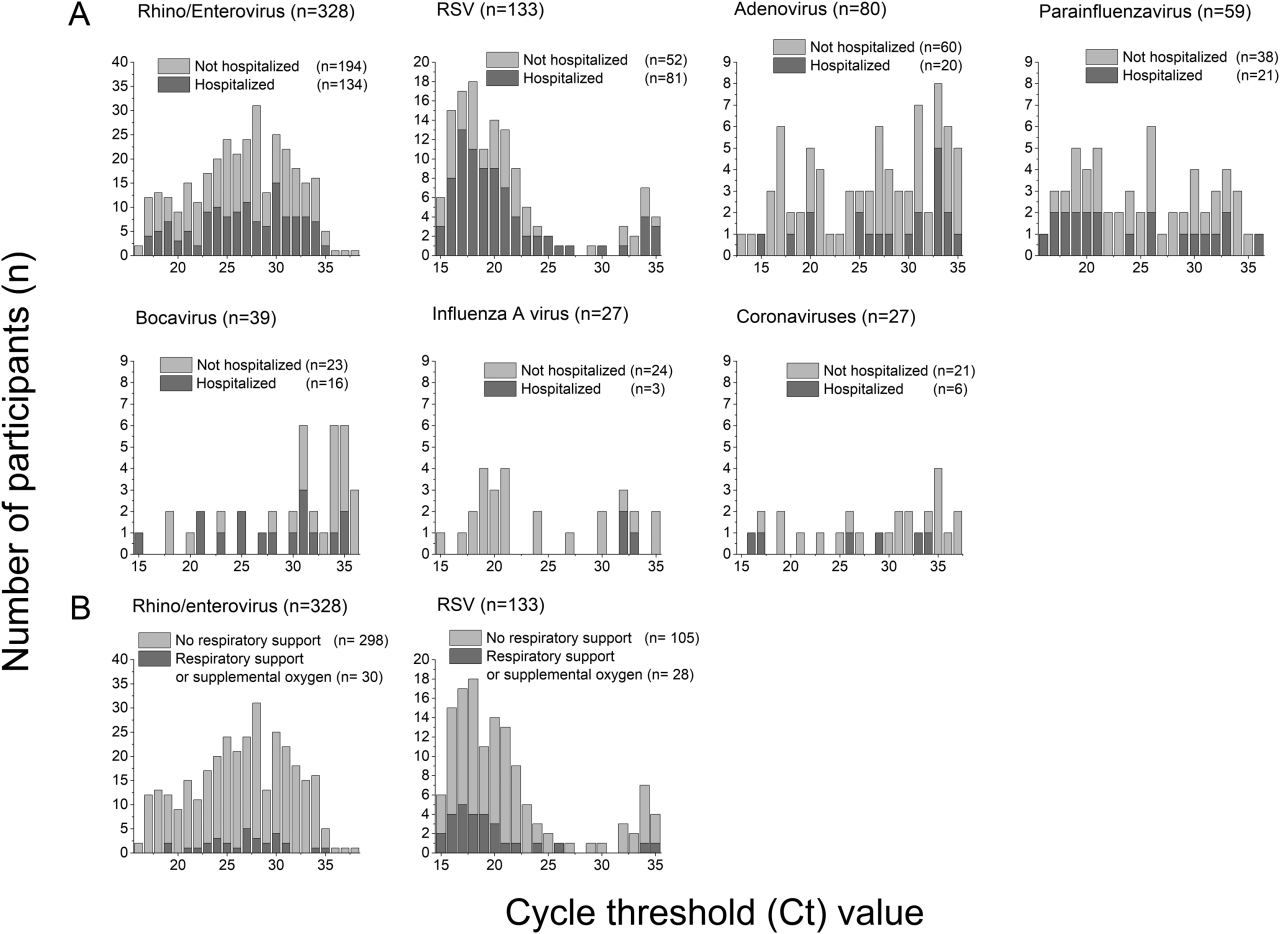
CT values and proportion of patients who were hospitalized or needed respiratory support. (A) Distribution of the CT values and proportion of patients who needed hospitalization. Dark gray bars represent children admitted to the hospital. (B) Distribution of the CT values and proportion of patients who needed respiratory support. Dark gray bars represent children who need respiratory support. Abbreviation: CT, cycle threshold.

The proportion of hospitalizations ranged from 11% (3/27) for those with influenza A virus to 61% (81/133) for those with RSV infection (Figure 1A). Children hospitalized with RSV infection were mainly infants under 3 months of age (44/81, 54%). Altogether 30 of 328 (9.1%) patients with rhino/enterovirus and 28 of 133 (21%) patients with RSV infection needed respiratory support or supplemental oxygen (Figure 1B). In total, 4 of 328 (1.2%) patients with rhino/enterovirus detection and 5 of 133 (3.8%) patients with RSV infection were admitted to the pediatric ICU either directly from the ED or later after hospitalization. When adjusted for age and sex, we observed no statistically significant associations between the CT values of respiratory viruses and the need for hospitalization, respiratory support, or intensive care (Table 1).

**Table 1.** Associations Between CT Value and Hospitalization, ICU Admission, or Need for Respiratory Support With Adjustment for Age and Sex

	Hospital Admission			ICU Admission			Respiratory Support/Supplemental Oxygen		
	aOR	95% CI	P-value	aOR	95% CI	P-value	aOR	95% CI	P-value
Rhino/enterovirus (n = 328)	1.02	0.97–1.07	0.43	1.17	0.91–1.49	0.22	1.01	0.93–1.09	0.84
RSV (n = 133)	0.999	0.93–1.07	0.97	0.95	0.77–1.19	0.67	0.96	0.87–1.06	0.41
Adenovirus (n = 80)	1.07	0.98–1.17	0.13	NA			NA		
Parainfluenza viruses (n = 59)	0.96	0.86–1.08	0.51	NA			NA		
Bocavirus (n = 39)	0.90	0.80–1.02	0.10	NA			NA		
Influenza A virus (n = 27)	1.47	0.86–2.50	0.16	NA			NA		
Coronaviruses (` = 27)	0.98	0.82–1.17	0.82	NA			NA		
Human metapneumovirus (n = 6)	0.88	0.56–1.37	0.56	NA			NA		

Abbreviations: aOR, adjusted odds ratio; CI, confidence interval; ICU, intensive care unit; NA, not available, since sample size did not enable analyses, CT, cycle threshold.

^a^Adjusted for age and sex.

## DISCUSSION

In this cohort study, the lower CT value of a respiratory virus, a semi-quantitative measurement to reflect a higher virus genome load, was not related to the need for hospitalization, respiratory support, or pediatric intensive care.

The CT values of respiratory viruses other than RSV have previously shown limited clinical benefit [5, 6]. In the previous studies with 300–500 patients with RSV infections, low CT values were associated with hospitalization [5, 6]. In this study of 133 patients with RSV detection no such association was found. The children hospitalized with RSV infection in the present study were mainly young infants who needed follow-up due to increased risk of apnea or breathing difficulties.

In this study, the CT values of rhino/enterovirus or RSV at the pediatric ED showed no association with the need for intensive care at the ED or later during hospitalization. In a previous study of 2400 children with rhinovirus, higher viral loads increased the risk of needing intensive care [3]. Since number of the patients with acute respiratory infection who needed transfer to intensive care was low, it may require a larger sample size to detect the association between CT and need for transfer to ICU. However, not all studies have shown associations between CT values and the need for intensive care in patients with rhinovirus or RSV detection [11]. In addition, previous studies indicate that higher viral loads have not been associated with respiratory support in children with rhinovirus infections [3, 6] which is in line with our results.

Ideally, the prediction of severe clinical outcomes would already be possible upon arrival at the ED, based on the CT values received from multiplex POC diagnostics. This is one of the first studies in which nasopharyngeal samples were analyzed as a POC test in a pediatric ED which makes the results generalizable to such testing in acute care. The study consisted of a representative sample of acutely ill children including patients with comorbidities and children admitted to intensive care. The clinicians in the ED were unaware of the CT values, although the viral detection results were available. All tested respiratory viruses were circulating during the study period with a comparable respiratory virus activity as in the preceding years [12]. The use of a multiplex PCR panel provides detailed information on co-detections. There were virtually no data lacking for clinical outcomes. The same diagnostic device was used throughout the study. We trained all nurses working in the ED for sample collection and the use of diagnostic devices before the study commenced.

Our study has some limitations. The diagnostic platform used for this study did not separate subtypes for RSV or rhino and enteroviruses. We did not perform pairwise comparisons between dry and diluted samples. Sample size did not enable analyses on ICU admissions and respiratory support for other viruses than rhinovirus and RSV. It is often unclear which virus dominates in co-infections. The detection of a respiratory virus may reflect an asymptomatic infection or prolonged viral shedding after clinical resolution of symptoms. We obtained a single nasopharyngeal swab and the exact time interval between the onset of symptoms and the nasopharyngeal swab was not available for analysis. However, obtaining repeated daily swabs may not be reasonable.

In conclusion, nasopharyngeal respiratory viral load, estimated by PCR CT values, was not associated with the need for hospitalization, respiratory support, or admission to the ICU. Based on this study, CT values are likely not useful in guiding clinical decision-making in acutely ill children.

## Supplementary Material

piad071_suppl_Supplementary_Tables_S1

## Notes

***Financial support*.** This work was supported by the Stiftelsen Alma and K.A. Snellman Foundation; The Finnish Medical Foundation; Finnish Pediatric Research Foundation; and University of Oulu Graduate School to Suvi Mattila, and Government Funding for Clinical Research at Oulu University Hospital. The funding organizations did not participate in the design and conduct of the study, collection, management, analysis, and interpretation of the data, preparation, review, or approval of the manuscript or decision to submit the manuscript for publication.

**Potential conflicts of interest.** All the authors declare that they have no conflicts of interest to disclose.

## Notes

**Main points:** In this cohort study of 800 children with fever or respiratory symptoms, the cycle threshold values of point-of-care multiplex polymerase chain reaction testing for respiratory viruses, inversely reflecting the virus genome load, were not associated with clinical outcomes.
