# Supplementary material for: Clinical Outcomes of Acutely Ill Children According to Cycle Threshold Values of Respiratory Viruses Detected by Multiplex PCR Testing
Source: J Pediatric Infect Dis Soc. 2023 Sep 20;12(10):549–52. doi: 10.1093/jpids/piad071 (PMC10977037; doi:10.1093/jpids/piad071)
Supplement: piad071_suppl_Supplementary_Tables_S1 [file piad071_suppl_supplementary_tables_s1.docx]

Supplemental Table 1. Baseline characteristics

| \|  \| All (n=800) \| Any organism detected (n=594) \| No organism detected (n=206) \| \| --- \| --- \| --- \| --- \| \| Age (SD) \| 3.0 (3.6) \| 2.6 (3.2) \| 4.1 (4.6) \| \| Girls - no. (%) \| 356 (44.5) \| 263 (44.3) \| 93 (45.1) \| \| Hospitalized – no. (%) \| 334 (41.8) \| 241 (40.6) \| 93 (45.1) \| \| Any antibiotic in the ED – no. (%) \| 217 (27.1) \| 147 (24.7) \| 70 (34.0) \| \| Crp in the ED^a^ (SD) \| 43.7 (61.9) \| 40.5 (59.8) \| 51.8 (66.4) \| \| Underlying medical conditions – no. (%) \|  \|  \|  \| \| Any underlying medical condition – no. (%) \| 200 (25) \| 142 (23.9) \| 58 (28.2) \| \| Asthma \| 25 (3.1) \| 24 (4.0) \| 1 (0.5) \| \| Diabetes \| 3 (0.4) \| 2 (0.3) \| 1 (0.5) \| \| Intellectual disability \| 16 (2.0) \| 9 (1.5) \| 7 (3.4) \| \| Down’s syndrome \| 8 (1.0) \| 6 (1.0) \| 2 (1.0) \| \| Preterm (<32) \| 15 (1.9) \| 11 (1.9) \| 4 (1.9) \| \| Preterm (32+0–36+6) \| 20 (2.5) \| 16 (2.7) \| 4 (1.9) \| \| Heart disease \| 11 (1.4) \| 8 (1.3) \| 3 (1.5) \| \| Primary immunodeficiency \| 2 (0.3) \| 1 (0.2) \| 1 (0.5) \| \| Epilepsy \| 10 (1.3) \| 6 (1.0) \| 4 (1.9) \| \| Congenital malformation \| 26 (3.3) \| 15 (2.5) \| 11 (5.3) \| \| Organ transplant \| 3 (0.4) \| 1 (0.2) \| 2 (1.0) \| \| Pulmonary disease \| 4 (0.5) \| 3 (0.5) \| 1 (0.5) \| |
| --- | --- | --- | --- | --- | --- | --- | --- | --- | --- | --- | --- | --- | --- | --- | --- | --- | --- | --- | --- | --- | --- | --- | --- | --- | --- | --- | --- | --- | --- | --- | --- | --- | --- | --- | --- | --- | --- | --- | --- | --- | --- | --- | --- | --- | --- | --- | --- | --- | --- | --- | --- | --- | --- | --- | --- | --- | --- | --- | --- | --- | --- | --- | --- | --- | --- | --- | --- | --- | --- | --- | --- | --- | --- | --- | --- | --- | --- | --- | --- | --- |

Abbreviations: CRP, C-reactive protein; ED, Emergency Department; SD, standard deviation

^a^Within 1 day after study entry
